# Clinical and maternal factors related to father’s involvement in pregnancy: a cross-sectional study

**DOI:** 10.1590/0034-7167-2025-0169

**Published:** 2026-07-31

**Authors:** Hillary Bastos Vasconcelos Rodrigues, Victórya Suéllen Maciel Abreu, Bruna Barroso de Freitas, Isaque Lima de Farias, Sarah de Sousa Carvalho, Herla Maria Furtado Jorge, Samila Gomes Ribeiro, Priscila de Souza Aquino

**Affiliations:** I Universidade Federal do Ceará. Fortaleza, Ceará, Brazil; II Universidade Federal do Piauí. Teresina, Piauí, Brazil

**Keywords:** Fathers, Nursing, Paternal Behavior, Pregnant Women, Prenatal Care., Pai, Enfermagem, Comportamento Paterno, Gestantes, Cuidado Pré-Natal., Padre, Enfermería, Conducta Paterna, Personas Embarazadas, Atención Prenatal.

## Abstract

**Objectives:**

to verify the paternal involvement in pregnancy according to maternal and clinical factors.

**Methods:**

cross-sectional study conducted in two primary health care units in Fortaleza, Brazil, during prenatal consultations with 130 women between May 2022 and March 2023. Participants completed the Paternal Involvement in Pregnancy Inventory and a form on sociodemographic, obstetric, and prenatal characteristics. Statistical analyses included the Shapiro-Wilk test, Mann-Whitney U test, and Spearman’s correlation.

**Results:**

half of the fathers never accompanied women to medical appointments, although most were always involved in other aspects. Paternal involvement was associated with paid employment (p = 0.049), family income (p = 0.022), living with a partner (p < 0.001), children at home (p = 0.035), and number of pregnancies (p = 0.035).

**Conclusions:**

greater paternal involvement was observed among employed women, living with a partner, with fewer children at home, and up to two pregnancies, while lower income was associated with less involvement.

## RESUMO

**Objetivos:**

verificar o envolvimento paterno na gestação de acordo com fatores maternos e clínicos.

**Métodos:**

estudo transversal realizado em duas unidades de atenção primária à saúde em Fortaleza, Brasil, durante consultas de pré-natal com 130 mulheres entre maio de 2022 e março de 2023. As participantes responderam ao *Paternal Involvement in Pregnancy Inventory* e a um formulário sobre características sociodemográficas, obstétricas e do pré-natal. As análises estatísticas incluíram o teste de Shapiro-Wilk, o teste U de Mann-Whitney e a correlação de Spearman.

**Resultados:**

metade dos pais nunca acompanhou as mulheres às consultas médicas, embora a maioria estivesse sempre envolvida em outros aspectos. O envolvimento paterno esteve associado ao trabalho remunerado (p = 0,049), à renda familiar (p = 0,022), a morar com a parceira (p < 0,001), à presença de filhos no domicílio (p = 0,035) e ao número de gestações (p = 0,035).

**Conclusões:**

observou-se maior envolvimento paterno entre mulheres empregadas, que viviam com o parceiro, tinham menos filhos em casa e até duas gestações, enquanto uma menor renda foi associada a menor envolvimento.

## RESUMEN

**Objetivos:**

verificar la participación paterna durante el embarazo según factores maternos y clínicos.

**Métodos:**

estudio transversal realizado en dos unidades de atención primaria de salud en Fortaleza, Brasil, durante consultas prenatales con 130 mujeres entre mayo de 2022 y marzo de 2023. Las participantes completaron el *Paternal Involvement in Pregnancy Inventory* y un formulario sobre características sociodemográficas, obstétricas y prenatales. Los análisis estadísticos incluyeron la prueba de Shapiro-Wilk, la prueba de Mann-Whitney U y la correlación de Spearman.

**Resultados:**

la mitad de los padres nunca acompañó a las mujeres a las consultas médicas, aunque la mayoría siempre participó en otros aspectos. La participación paterna se asoció con el empleo remunerado (p = 0,049), los ingresos familiares (p = 0,022), la convivencia con la pareja (p < 0,001), los hijos en el hogar (p = 0,035) y el número de embarazos (p = 0,035).

**Conclusiones:**

se observó una mayor participación paterna entre las mujeres empleadas, que convivían con la pareja, tenían menos hijos en el hogar y hasta dos embarazos, mientras que los ingresos más bajos se asociaron con menor participación.

## INTRODUCTION

Pregnancy is an important milestone in a woman’s life, involving various physical, hormonal, physiological, and psychological changes^(1)^. In this sense, health care is essential throughout the entire period. Furthermore, social support during pregnancy is of great importance, as there is evidence that its absence is associated with mental health problems in pregnant women-such as anxiety, depression, and self-harm^(2)^.

The partner, as part of the support network, plays an indispensable role in providing support and coping with the effects of pregnancy. This involvement directly benefits the mother, contributes to the couple’s relationship, and strengthens the father-mother-child triad^(3)^.

The World Health Organization’s (WHO) recommendations on prenatal care for a positive pregnancy experience and the Brazilian Ministry of Health’s emphasize the critical role of health professionals in promoting paternal involvement, from pre-pregnancy planning to the entire child development process, with a focus on promoting positive family formation^(4,5)^.

Various forms of demonstrating paternal involvement emerge through acknowledging the reality of fatherhood, witnessing ultrasounds, and perceiving fetal movements, leading to increased emotional engagement with the pregnancy and fetus. In addition, a commitment to be present, such as attending prenatal appointments and spending more time with the pregnant partner, is noteworthy^(6)^.

Several studies indicate a variety of benefits associated with paternal involvement during pregnancy, which include from the construction and maturation of the paternal role to positive impacts on the mother-baby binomial and child development, especially in the first five years of life^(7)^. In addition, a survey conducted in the United States identified that 86.3% of pregnant women with partners started prenatal care in the first trimester, which contributes significantly to the proper gestational development and to the reduction of barriers in access to health services^(8)^. Such evidence reinforces the importance of strengthening paternal participation throughout pregnancy.

The main reasons for paternal absence from prenatal care include lack of knowledge, paternal exclusion from consultations, disinterest, and distance in the relationship between the accompanying partner and the professional team^(9)^.

In this context, it is essential to understand how maternal and clinical factors can influence the level of paternal involvement during pregnancy.

On the national scene, studies on paternal involvement during pregnancy indicate that it has been treated as an adjunct, with priority given to the mother-infant binomial in prenatal care. This approach highlights the existence of barriers, especially of assistance order, which hinder the effective integration of the father figure in this period^(10)^.

In a convergent way, international researches corroborate this perspective by pointing out that the health services, in different contexts, still show little inclusion regarding the father’s participation during pregnancy and associated care^(6,11)^. Thus, understanding the factors that condition paternal integration in pregnancy is essential for the implementation of strategies that favor their role as protagonists in care.

Parental support and involvement are determinants of family well-being; However, its consolidation remains limited by cultural and social barriers that restrict the full participation of men in care^(12)^. Therefore, there is a need to understand the paternal involvement during the prenatal period on the clinical and maternal factors that can influence this participation, to improve the care offered, including the father figure in this stage of life.

Therefore, this study aims to contribute to the national scenario of scientific production in nursing, impacting on a better care offered, since it verifies in its construct the paternal involvement in pregnancy according to maternal and clinical factors, since it is observed, yet, a care scenario that highlights gaps in promoting integration and active participation of the father figure in prenatal care^(6,10)^. It is considered, therefore, that such evidence serve as a basis for rethinking practices and public policies, in order to promote strategies that ensure paternal protagonism as a fundamental element for the quality of care and for the strengthening of family ties since pregnancy.

## OBJECTIVES

To verify the paternal involvement in pregnancy according to maternal and clinical factors.

## METHODS

### Ethical aspects

The study was approved by the Research Ethics Committee of the Federal University of Ceará, with protocol number 5.325.733. The principles of research involving human subjects, as outlined in Resolution No. 466/12 - CNS/Brazil, were strictly followed.

### Type of study, period and location of study

This cross-sectional observational study was guided by the STROBE (Strengthening the Reporting of Observational Studies in Epidemiology) checklist^(13)^. Data collection took place from May 2022 to March 2023, involving pregnant women during their prenatal consultations at two primary health care units in the city of Fortaleza, Ceará, Brazil. The two primary health care units were selected for convenience, belonging to the area with similar sociodemographic characteristics and belonging to the area of the nursing program at the Federal University of Ceara.

### Study Sample

The study population consisted of all pregnant women receiving prenatal care in the selected units, which is equivalent to 164. Convenience sampling was used, recruiting participants based on pre-screening of medical records, with the following inclusion criteria: women at low obstetric risk, aged 18 years or older, and with a gestational age of at least 20 weeks. The exclusion criteria were: pregnant women with hearing impairments that prevented communication for the application of the instruments. Thus, a total of 130 expectant mothers participated in the study.

### Study protocol

Data were collected using a researcher-designed sociodemographic and obstetric characterization form and the Father’s Involvement during Pregnancy Inventory. Prior to formal data collection, a pilot test was conducted with four pregnant women, these did not comprise the final study sample, to assess instrument comprehension, administration time, and potential adaptations.

The Father’s Involvement during Pregnancy Inventory assesses paternal involvement and commitment during pregnancy through five items rated on a Likert scale from 1 to 5. This is a scale developed by Rodrigues, et al. (2010), designed to measure the degree of paternal involvement during pregnancy in the context of Portuguese families, in which items 1 and 2 assess prenatal involvement, while items 3 to 5 assess pregnancy support. A higher calculated score on this instrument indicates greater paternal involvement during pregnancy. It has a Cronbach’s alpha of 0.698 and the final score ranges from 5 to 25^(14)^.

The instrument used in this study has not been validated for the context of Brazilian families. However, linguistic adaptation was carried out beforehand, with three obstetric nurses, with the replacement of Portuguese terms with expressions and words that are more faithful to the linguistic context of the Brazilian Northeast.

Maternal variables refer to the mother’s sociodemographic and family data, such as: maternal age, maternal ethnicity, maternal education, paid employment during pregnancy, family income, maternal religion, number of children living together, age of youngest child, cohabitation with partner.

There are clinical variables: number of pregnancies, nulliparity, previous abortions, planned pregnancy, desired pregnancy, gestational age, number of prenatal consultations, complications in previous pregnancies, complications in the current pregnancy.

It is noteworthy that the maternal ethnicity variable considers any woman who self-identifies as black or mixed race, according to the Brazilian Law 12.288 of July 20, 2010, which establishes the Racial Equality Statute^(15)^.

The outcome variable examined was paternal involvement during pregnancy, based on scores from the Father’s Involvement during Pregnancy Inventory by Rodrigues et al. (2010).

### Analysis of results and statistics

The data collected were tabulated and analyzed using IBM^®^ SPSS^®^ Statistics, version 22.0. Descriptive analysis was performed, including the Shapiro-Wilk test to assess normality (indicating a non-normal distribution), calculation of medians, minimum and maximum values, and interquartile range for numerical variables. The Mann-Whitney U test was used to compare medians between groups, with statistical significance set at p < 0.05. In addition, Spearman’s correlation test was performed for numeric variables.

## RESULTS

A total of 130 pregnant women were included in the study. The median maternal age was 26 years, with an interquartile range (IQR) of 8. The majority were of mixed ethnicity (n=95; 73.1%) and religious affiliation (n=102; 78.5%).

The majority of pregnant women were not employed during pregnancy (n=67; 51.5%), with a median family income of R$ 1,212.00 Brazilian Reais (IQR=1,415.00), which was the minimum wage at the time.

In addition, the majority were in stable relationships or married (n=89; 68.5%) and lived with their partners (n=108; 83.1%). The median number of children living together was one (IQR=1), while the median age of the youngest child was 33.5 months (2 years, 9 months, and 15 days) (IQR=60).

Regarding the clinical-obstetric background, there was a prevalence of nulliparous women (n=56; 43.1%) and primiparous women (n=39; 30.0%). The maximum number of pregnancies found was six, including interrupted pregnancies, with a median of two pregnancies (IQR=2). In addition, the maximum number of previous deliveries was five, with a median of one (IQR=2). Regarding previous miscarriages, 99 women (76.2%) denied any previous experience.

Regarding the current pregnancy, there was a prevalence of unplanned pregnancies (n=87; 66.9%), although there was a higher frequency of wanted pregnancies (n=119; 91.5%).

Table 1 shows the frequency of parental participation in each item of the inventory.

**Table 1:** Frequency of responses regarding items on the Father’s Involvement during Pregnancy Inventory among pregnant women interviewed, Fortaleza, Ceará, Brazil, 2023, N=130

Inventory items	Father’s Involvement during Pregnancy Inventory	
	n	%
**Accompany her to her pregnancy checkups**		
Never	65	50.0
Rarely	12	9.2
Sometimes	15	11.5
Almost always	16	12.3
Always	22	16.9
**Accompany her to her ultrasound appointments**		
Never	35	26.9
Rarely	11	8.5
Sometimes	17	13.1
Almost always	12	9.2
Always	55	42.3
**Feel the baby’s movements**		
Never	14	10.8
Rarely	4	3.1
Sometimes	8	6.2
Almost always	11	8.5
Always	93	71.5
**Participate in preparing the baby’s room**		
Never	17	13.1
Rarely	4	3.1
Sometimes	5	3.8
Almost always	10	7.7
Always	94	72.3
**Participate in preparing the baby’s layette**		
Never	16	12.3
Rarely	6	4.6
Sometimes	5	3.8
Almost always	7	5.4
Always	96	73.8

Regarding parental involvement, it was observed that half never accompany pregnant women to appointments, while the other half accompany them with varying regularity. In other aspects of involvement, there was also variation in frequency, but the prevailing percentage in all cases was that they were always involved in that activity.

Median scores on the Father’s Involvement during Pregnancy Inventory were compared based on maternal sociodemographic factors, as shown in Table 2.

**Table 2:** Comparison of the medians of scores from the Father’s Involvement during Pregnancy Inventory according to maternal sociodemographic variables, Fortaleza, Ceará, Brazil, 2023, N=130^*^

Socio-demographic variables	Father’s Involvement during Pregnancy Inventory	
	Median (IQR)	*p* value
**Age of mother**		
Up to 35 years	19.0 (7.0)	0.231
35 years or older	21.0 (4.0)	
**Maternal education**		
Up to 9 years of study	18.5 (5.0)	0.090
More than 9 years of study	21.0 (6.0)	
**Maternal ethnicity**		
Black	21.0 (6.0)	0.074
Non-black	19.0 (8.0)	
**Maternal religion**		
Yes	20.5 (5.0)	0.998
No	19.0 (10.0)	
**Paid employment during pregnancy**		
Yes No	21.0 (6.0) 19.0 (7.0)	0.049^*^
**Family income**		
Up to 1 minimum wage	19.0 (11.0)	0.022^*^
Above 1 minimum wage	21.0 (6.0)	
**Lives with partner**		
Yes	21.0 (6.0)	**>0.001**
No	8.0 (14.0)	
**Co-residing children**		
Up to 2	20.5 (6.0)	0.035^*^
More than 2	15.5 (7.0)	
**Age of youngest child**		
Up to 1 year old	19.0 (4.0)	0.390
Over 1 year old	21.0 (7.0)	

* *Mann-Whitney U test.*

As shown in Table 2, the comparisons revealed significant differences in the following maternal aspects. Women who worked during pregnancy (p=0.049) had higher median scores for paternal involvement compared to those who did not work. Those with a family income of up to one minimum wage (p=0.022) had lower median scores for paternal involvement during pregnancy compared to those with higher family incomes.

In addition, women who lived with their partner (p>0.001) had higher median scores for paternal involvement compared to those who did not live with their partner. Also, women living with up to two children (p=0.035) had higher median scores for paternal involvement compared to those living with more than two children.

Regarding the clinical-obstetric aspects, a comparison of the median scores of the Father’s Involvement during Pregnancy Inventory was performed, as shown in Table 3.

**Table 3:** Comparison of the medians of scores from the Father’s Involvement during Pregnancy Inventory, according to maternal clinical factors, Fortaleza, Ceará, Brazil, 2023, N=130^*^

Maternal clinical factors	Father’s Involvement during Pregnancy Inventory	
	Median (IQR)	*p* value
**Number of pregnancies, including current**		
Up to 2	21.0 (6.0)	**0.035** ^*^
More than 2	19.0 (5.0)	
**Nulliparous**		
Yes	20.0 (8.0)	0.553
No	19.0 (5.0)	
**Previous miscarriage**		
Yes	19.0 (12.0)	0.246
No	21.0 (6.0)	
**Planned pregnancy**		
Yes	19.0 (6.0)	0.249
No	20.0 (8.0)	
**Desired pregnancy**		
Yes	20.0 (6.0)	0.129
No	17.0 (11.0)	
**Gestational age**		
Second trimester	20.5 (6.0)	0.907
Third trimester	19.0 (7.0)	
**Number of prenatal visits**		
Up to 5	19.0 (6.0)	0.599
More than 5	21.0 (7.0)	
**Intercurrences in previous pregnancy**		
Yes	21.5 (9.0)	0.109
No	19.0 (4.0)	
**Intercurrences in the current pregnancy**		
Yes	20.0 (5.0)	0.559
No	19.0 (7.0)	

* *Mann-Whitney U test.*

With regard to Table 3, the comparisons revealed statistically significant differences regarding the number of pregnancies (p=0.035). In this analysis, women who had up to two pregnancies had higher median paternal involvement scores than those with more than two pregnancies.

In addition, correlations were established between maternal sociodemographic and gynecologic-obstetric variables and median scores on the Father’s Involvement during Pregnancy Inventory, as shown in Table 4.

**Table 4:** Correlation between sociodemographic and gynecological-obstetric antecedents with the median of the Father’s Involvement during Pregnancy Inventory among interviewed pregnant women, Fortaleza, Ceará, Brazil, 2023, N=130^*^

Sociodemographic and Gynecological-Obstetric Variables	Father’s Involvement during Pregnancy Inventory	
	r	*p* value
Maternal age	0.048	0.588
Family income	0.246	**0.005**
Maternal education	0.146	0.098
Number of residents in the house	-0.161	0.68
Number of cohabiting children	- 0.089	0.313
Age of the youngest child	0.135	0.253
Number of pregnancies	-0.112	0.206
Number of births	-0.093	0.295
Number of prenatal consultations	0.004	0.965
Gestational age	-0.019	0.828

* *Spearman Correlation Test.*

As analyzed in Table 4, the variable “family income” showed a directly proportional correlation with the outcome, indicating that the lower the income, the less paternal involvement during pregnancy. This finding is consistent with the results in Table 2.

## DISCUSSION

Based on the findings, it was possible to observe that fathers are more involved during pregnancy when it comes to preparing the baby’s trousseau, the baby’s room and feeling the baby’s movements. In this regard, a scoping study highlights that the news of pregnancy raises the father’s awareness of the need to prepare the environment for the child, assuming as one of his main roles that of family provider^(16)^. From this perspective, there are influences from the roles attributed to gender with regard to financial provision by parents^(17)^. In addition, the act of touching the pregnant woman’s belly is perceived by fathers as a way of establishing a bond with their child^(16)^. In this sense, a survey of 600 men showed that stroking the belly as a form of prenatal interaction is one of the ways men prefer^(18)^.

An analysis of the results of the inventory items shows that the item concerning the father’s frequency of prenatal visits was the only one that was predominantly answered as “never”, indicating a greater absence of parental involvement in this phase, in opposition to the paternal presence in ultrasound consultations, where 42.3% always reported participating. In line with this, research conducted in South Africa found that 32.5% of partners attended antenatal visits, while 42.4% attended ultrasound appointments^(11)^. In this sense, a qualitative study shows that parents feel only with the role of support during prenatal consultations, among the parental reasons for not participating in prenatal consultations are inflexible work arrangements, waiting time for queries and cancellation of these^(19)^. In contrast, a systematic review study shows that parents see ultrasound as an opportunity to get to know their child^(20)^. Thus, there is a need to emphasize the importance of parental care during prenatal consultations.

In view of this, a systematic review of paternal involvement in pregnancy and childbirth in Africa found that fathers often felt alienated when it came to participating in maternal health services. This is because the structure of care was directed exclusively at women, ranging from physical spaces to obtaining targeted information^(21)^.

In addition, it was observed that women who did not live with their partner had less paternal involvement in the pregnancy than those who did. One dimension of this bond with the pregnant woman concerns instrumental support, which is translated into the completion of household activities, as this participation results in free time for the pregnant woman to rest, relax, and attend to other pending tasks^(22,23)^. On the other hand, there is evidence that physical distance after childbirth leads to negative changes in maternal mood, with consequent harm to the child, as the father plays an important role in the child’s psychosocial development, with maternal parenting mediating this distant relationship^(24)^.

Regarding family income and paid employment during pregnancy, a negatively influence of low economic conditions on paternal involvement was observed. In line with this, a longitudinal German study found that fathers’ primary concerns during pregnancy are related to the financial even, with these concerns being more pronounced among fathers with lower incomes. This is likely due to the fact that paternal employment often represents the main source of household income, placing greater pressure on the father figure^(25)^. In this context, other studies indicate that financial instability inhibits paternal involvement, as fathers tend to prioritize their income-generating activities, thereby limiting their participation during pregnancy^(26,27)^; lack of time due to work-related commitments is another factor associated with reduced involvement^(17)^. This highlights the significance fathers attribute to this social determinant of health, which influences their level of engagement throughout the gestational period.

In this study, women with a higher number of pregnancies and cohabiting children had less paternal involvement in pregnancy. Similarly, a qualitative study carried out in Nigeria observed that as the number of children increases, paternal support during pregnancy may decrease due to the need to care for other children and the associated costs^(28)^. Corroborating these findings, a study conducted in Tanzania identified family size as a factor influencing men’s involvement during pregnancy, with the responsibility of caring for other children during their partners’ absence acting as a barrier to their involvement^(17)^. Furthermore, an integrative review showed that first-time fathers were more likely to be involved compared to those who already had multiple children^(29)^.

For its part, higher levels of education and better financial conditions are associated with more active paternal involvement in child rearing and marital satisfaction^(30)^. Therefore, it can be inferred that fathers with greater financial stability may be more involved in child rearing because they have fewer worries about providing for the household and family comfort.

### Study limitations

This study has limitations in terms of its representativeness, as the sample consisted only of pregnant women from public health units, with no information available on those using private services or those in high-risk situations. Furthermore, the lack of a formal process of cross-cultural validation of the instrument used is highlighted as a limitation.

### Contributions to the Field of Nursing and Public Health

Identifying factors related to paternal involvement in pregnancy is important for training nursing professionals with transformative potential, capable of identifying challenges to paternal involvement during prenatal care and intervening in a positive manner. Therefore, it is important to investigate the existing barriers to paternal involvement in pregnancy in clinical practice and train nursing professionals to provide more comprehensive and humanized care, since they are professionals with a significant role in prenatal care.

In this context, it is essential to promote future investigations that explore paternal determinants, as well as the perspectives, experiences, and barriers perceived by fathers themselves in relation to pregnancy and fatherhood.

## CONCLUSIONS

Given the above, there is greater paternal involvement in pregnancy among women who work during pregnancy, who live with their partner, who have fewer children living with them, and who have a history of up to two pregnancies (including the current one). On the other hand, there was less paternal involvement among women with lower family income. The analysis of these maternal and clinical factors highlights the complexity surrounding paternal involvement during pregnancy.

## Data Availability

The research data are available in a repository: https://doi.org/10.48331/SCIELODATA.JHKMHB.
